# 1,357 AI medical devices cleared, 3 actually tested on patient outcomes

**DOI:** 10.1371/journal.pdig.0001597

**Published:** 2026-08-19

**Authors:** Rawan Abulibdeh, Sebastián Andrés Cajas Ordóñez, Leo Anthony Celi, Rahul Gorijavolu, Nura Izath, Torleif Markussen Lunde

**Affiliations:** 1 Department of Electrical and Computer Engineering, University of Toronto, Ontario, Canada; 2 MIT Critical Data, Massachusetts Institute of Technology, Cambridge, Massachusetts, United States of America; 3 Laboratory for Computational Physiology, Massachusetts Institute of Technology, Cambridge, Massachusetts, United States of America; 4 Division of Pulmonary, Critical Care and Sleep Medicine, Beth Israel Deaconess Medical Center, Boston, Massachusetts, United States of America; 5 Department of Biostatistics, Harvard T.H. Chan School of Public Health, Boston, Massachusetts, United States of America; 6 School of Medicine, Johns Hopkins University, Baltimore, Maryland, United States of America; 7 Whiting School of Engineering, Johns Hopkins University, Baltimore, Maryland, United States of America; 8 AI for Responsible, Generalizable, and Open Surgical Research Group, Baltimore, Maryland, United States of America; 9 Mbarara University of Science and Technology Data Science Research Hub (MUDSReH), Mbarara, Uganda; 10 Vestlandets Innovasjonsselskap, Postboks, Bergen, Norway; 11 University of Bergen, Bergen, Norway; Yonsei University College of Medicine, KOREA, REPUBLIC OF

## Abstract

Artificial intelligence (AI) tools are entering clinical practice at unprecedented speed. 1,357 AI/ML-enabled medical devices have received U.S. FDA clearance or approval, yet their impact on patient outcomes remains largely untested. We conducted a systematic analysis of all FDA-cleared AI/ML-enabled medical devices through December 5, 2025 using the FDA device database and the ACR Data Science Institute catalogue, with linked searches of ClinicalTrials.gov and PubMed to identify registered trials and publications. Of 1,357 cleared AI devices, only 34 (2.5%) were linked to registered prospective trials, 12 (0.9%) posted results, 12 (0.9%) had peer-reviewed publications, and only 3 (0.2%) evaluated patient-centered outcomes such as mortality, morbidity, or readmissions. Most studies (62%) employed observational designs with small, homogenous cohorts, limited subgroup analyses, and frequent exclusion of vulnerable populations. Structural barriers (including misaligned financial incentives, reliance on predicate-based regulatory pathways, and logistical challenges of multi-center trials) discourage rigorous evaluation. Internationally, FDA clearance often functions as a gateway for global deployment, raising ethical concerns when under-validated tools are introduced into low- and middle-income countries without contextual validation or safeguards. Regulatory approval has outpaced clinical validation, creating an ecosystem where innovation advances without accountability. The finding that only 0.2% of cleared devices have undergone evaluation for patient-centered outcomes reveals a profound validation gap and points to the need for evidence standards capable of keeping pace with the speed of regulatory clearance. Readiness should no longer be defined by FDA clearance alone, but by demonstrated, durable, and equitable benefit to patients.

## Introduction

**Fig 1:**
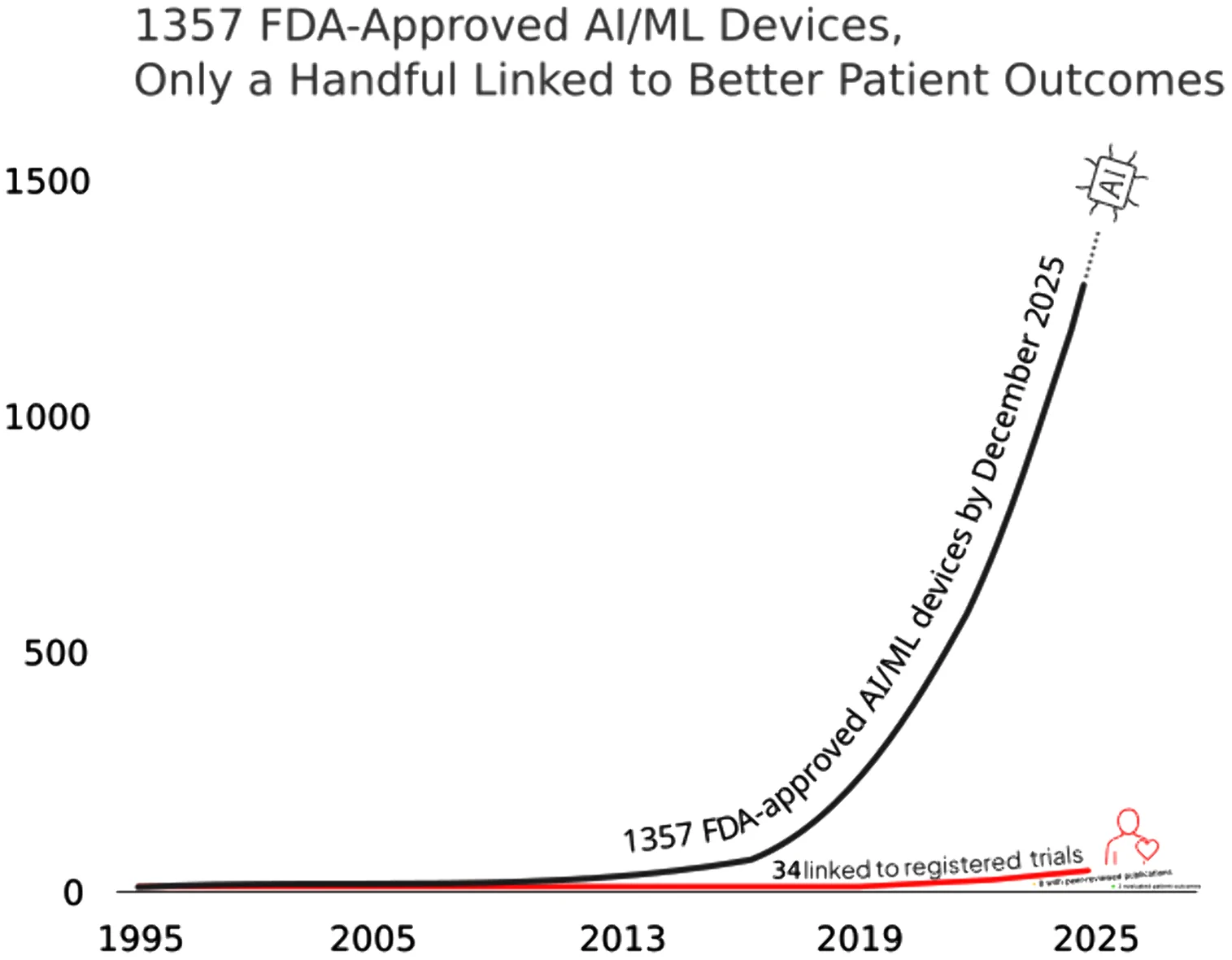
Steep contrast between the number of FDA-cleared AI/ML-enabled devices (1,357 as of December 5, 2025) and the handful linked to registered clinical trials or patient-centered outcomes. Despite thousands of regulatory clearances, only a tiny fraction have been evaluated for mortality, morbidity, or readmissions.

Artificial intelligence (AI) has entered healthcare at an unprecedented pace. 1,357 FDA-cleared AI-enabled devices are now marketed to clinicians [1], with radiology alone accounting for 78% (1,059/1,357) of cleared devices, followed by cardiovascular (9%), neurology (5%), and other specialties (8%) [2]. These systems already guide radiologists in interpreting mammograms [3,4], inform cardiologists about risk scores [5,6], and even influence surgical planning [7]. Clinical decisions increasingly depend on algorithmic outputs. Yet despite this rapid adoption, one question remains largely unanswered: do these tools actually improve patient outcomes? The regulatory framework for AI devices, dominated by the FDA’s 510(k) pathway, requires only that a new product demonstrate “substantial equivalence” to an existing one rather than prospective validation of clinical effectiveness [8]. Unlike the De Novo pathway, which requires independent demonstration of safety and effectiveness for novel devices, the 510(k) route allows evidence gaps to propagate through chains of predicate devices, many of which themselves lack rigorous clinical validation. This has created a paradox in which the market is crowded with AI tools, yet the evidence supporting patient-centered outcomes remains remarkably thin. The overwhelming majority of cleared devices lack linked prospective evaluation of patient-centered outcomes. The steep contrast between thousands of FDA-cleared devices and the handful with clinical validation is shown in Fig 1. This evidence attrition highlights the profound gap between regulatory authorization and clinically meaningful validation. Most devices instead rely on retrospective accuracy benchmarks, surrogate endpoints, or selective reporting, often using narrow datasets that fail to reflect the diversity of real-world populations [2,9,10].

The consequences of this evidence gap are tangible. This lack of rigorous validation carries risks once devices reach the clinic. An independent analysis of 950 FDA-cleared AI devices found that 6% were associated with recall events, and nearly half of those occurred within the first year of clearance [11]. While a device-level linkage of MAUDE recall data to our full cohort of 1,357 devices was beyond the scope of this evidence census, the recall rate reported by Lee et al. provides a conservative benchmark for post-market safety signals. High-profile failures further illustrate how algorithms can falter in real-world use. The Epic Sepsis Model, deployed in more than half of U.S. hospitals, failed to identify 67% of patients with sepsis while generating alerts for 18% of all hospitalized patients [12]. Similarly, IBM Watson for Oncology, adopted internationally after FDA clearance, achieved only 33% concordance with clinical decisions in Denmark and 12% for gastric cancer in China [13,14]. Drugs and implantable devices cannot be adopted without proof of clinical impact, yet AI tools are permitted into clinical practice without the same level of scrutiny [2,9]. Subgroup analyses by demographic and socioeconomic data are rarely conducted [9], leaving it unknown how these systems perform across diverse populations. This oversight not only threatens clinical confidence but risks entrenching disparities in care. The central issue in clinical AI is not whether algorithms can classify images or predict risk; it is whether they tangibly improve patient health. Without systematic reform, the gap between algorithmic capability and clinical evidence will continue to widen. This study aims to quantify the attrition of clinical evidence from FDA clearance to patient-outcome evaluation across all 1,357 AI/ML- enabled medical devices, while characterizing the design features, transparency practices, and equity considerations of the registered trials identified. Building on these findings, we offer two interpretive contributions: first, an analysis of the regulatory, economic, and logistical conditions that discourage rigorous prospective validation; and second, a staged roadmap of evidence standards intended to support more accountable AI device development. If AI in medicine is to be life-saving, it must first be life-tested.

## Methods

To evaluate the clinical impact of FDA-cleared AI devices, we systematically reviewed all 1,357 AI/ML-enabled medical devices cleared or approved by the U.S. FDA through December 5, 2025, using the FDA’s public device database [15] and the ACR-DSI AI Central catalogue [16]. Published outcome studies were identified via automated PubMed searches, while prospective trial registrations were retrieved from ClinicalTrials.gov [17] links embedded in FDA 510(k) summary pages and verified through manual review. To ensure transparency and reproducibility, we provide the full device list, trial identifiers, and extraction outputs in S1 File.

Study design and scope. This study was designed as a regulatory evidence census and structured evidence-mapping analysis of publicly available validation signals for FDA-cleared or approved AI/ML-enabled medical devices, rather than as a PRISMA-style meta-analysis of treatment effects. The objective was to characterize the presence, design features, and transparency of prospective clinical validation linked to authorized devices. Specifically, we sought to quantify: (1) what proportion of FDA-cleared AI devices have registered clinical trials, (2) the methodological characteristics and reporting completeness of those trials, and (3) equity considerations in trial design and enrollment.

Device identification and deduplication. We included all AI/ML-enabled medical devices listed in the FDA public database and the ACR Data Science Institute AI Central catalogue as of December 5, 2025. Devices were deduplicated using manufacturer name, device name, and FDA submission identifiers (510(k), De Novo, PMA). Throughout this manuscript, “FDA-cleared” refers to devices authorized via the 510(k) pathway, “FDA- approved” refers to those authorized via Premarket Approval (PMA), and “FDA-authorized” or “cleared or approved” is used as an umbrella term encompassing 510(k), De Novo, and PMA pathways. The vast majority of AI/ML-enabled devices have reached the market through the 510(k) pathway. A detailed breakdown by regulatory pathway is provided in S1 File.

Three-phase evidence mapping approach. Phase 1 consisted of an automated PubMed search using device name variants and manufacturer identifiers combined with predefined patient-outcome terms (mortality, morbidity, length of stay, readmission, complications). Phase 2 involved systematic extraction of ClinicalTrials.gov identifiers from FDA 510(k) summary pages using automated web scraping. Phase 3 linked identified NCT registrations to peer-reviewed publications using PubMed secondary identifier searches (NCT[si]) and abstract retrieval.

## Eligibility criteria and manual extraction

Trials were included if prospectively registered on ClinicalTrials.gov and linked to FDA-cleared AI/ML-enabled medical devices, regardless of publication or results posting status. For all eligible trials, the independent reviewers manually extracted study characteristics, sample size, demographic information, exclusion criteria, and developer type from ClinicalTrials.gov registry records. The reviewers came from clinical research and engineering backgrounds and were trained on the extraction protocol before data collection began. Data extraction was conducted using a standardized, pre-specified extraction form. Inter-rater reliability was not formally assessed using Cohen’s kappa or percentage agreement because the extracted variables were primarily objective and factual—such as sample size, registration status, and endpoint classification—rather than dependent on interpretive judgment. Extraction was not performed blinded to device manufacturer, as sponsor and developer type were among the variables explicitly extracted from registry records. When available, data were supplemented with information from peer-reviewed publications and posted trial results. Discrepancies were resolved by consensus.

Outcome classification. Patient-centered outcomes were defined a priori based on the trial’s primary endpoint. Primary (hard clinical) outcomes included mortality (e.g., survival, death rates) and major morbidity (e.g., stroke, myocardial infarction, serious adverse events). Hospitalizations and readmissions were considered patient-centered when designated as primary endpoints. Additional patient-centered endpoints included validated quality-of-life measures (e.g., SF-36, EQ-5D), functional status (activities of daily living, disability scores, return to work), and symptom burden affecting daily life. Process measures such as workflow efficiency or length of stay without direct linkage to clinical events were classified separately. Endpoints limited to diagnostic accuracy (e.g., sensitivity, specificity, AUC), technical performance metrics, physiological measurements without linkage to clinical outcomes, or specimen collection were classified as surrogate endpoints and not considered patient-centered for this analysis. Accordingly, outcomes were considered patient-centered only when the primary endpoint directly reflected tangible benefit to patients’ survival, safety, well-being, or functional capacity. Because the aim was to characterize validation presence and design features rather than to estimate pooled clinical effects, formal risk-of-bias scoring tools (e.g., ROBINS-I, QUADAS-AI) were not applied.

## The missing evidence for clinical impact

Across specialties, the proportion of cleared devices with registered prospective trials varied substantially (Table 1). Radiology, which accounts for 78% of cleared devices (1,059/1,357), has prospective trials for fewer than 1% of tools. Cardiovascular and neurology devices demonstrate modestly higher proportions of registered trials (9.5% and 9.7%, respectively), while anesthesiology, despite 22 cleared devices, has no registered prospective trials. The “Other” category, encompassing 88 devices across multiple smaller specialties, shows the highest relative trial rate (14.8%). Across all specialties, however, the overwhelming majority of trials concentrated on diagnostic (59%) or screening (21%) applications, with only three studies (9%) addressing therapeutic or treatment guidance (Table 2). These specialty-level differences should be interpreted descriptively and cautiously, given the small number of registered trials in several categories.

**Table 1:** FDA-cleared AI devices by specialty.

Field	Devices	With Trials	% With Trial
Radiology	1,059	3	0.3%
Cardiovascular	126	12	9.5%
Neurology	62	6	9.7%
Anesthesiology	22	0	0.0%
Other	88	13	14.8%

**Table 2:** Trial design and evidence characteristics among FDA-cleared AI devices with registered trials (*n* = 34).

Characteristic	n	%
***Study design***		
Prospective	30	88.2
Retrospective	4	11.8
Hybrid	0	0.0
Unknown	0	0.0
***Clinical task***		
Diagnostic	20	58.8
Screening	7	20.6
Therapeutic/treatment guidance	3	8.8
Other	4	11.8
***Sample size categories***		
< 100	9	26.5
100–500	16	47.1
> 500	9	26.5
Not reported	0	0.0
***Geographic distribution***		
U.S.-only	23	67.6
Single-country (non-U.S.)	0	0.0
International	10	29.4
Unknown	1	2.9
***Subgroup analyses reported***		
Any subgroup analysis reported	9	26.5
Not reported	23	67.6
Missing/unclear	2	5.9
***Developer type***		
Industry-sponsored	32	94.1
Academic-sponsored	1	2.9
Partnership	1	2.9
***Primary endpoint classification***		
Diagnostic accuracy/surrogate endpoints	28	82.4
Process endpoints (e.g., workflow efficiency, LOS without clinical event)	3	8.8
Patient-centered outcomes (mortality, major morbidity, functional status, QoL)	3	8.8

**Fig 2:**
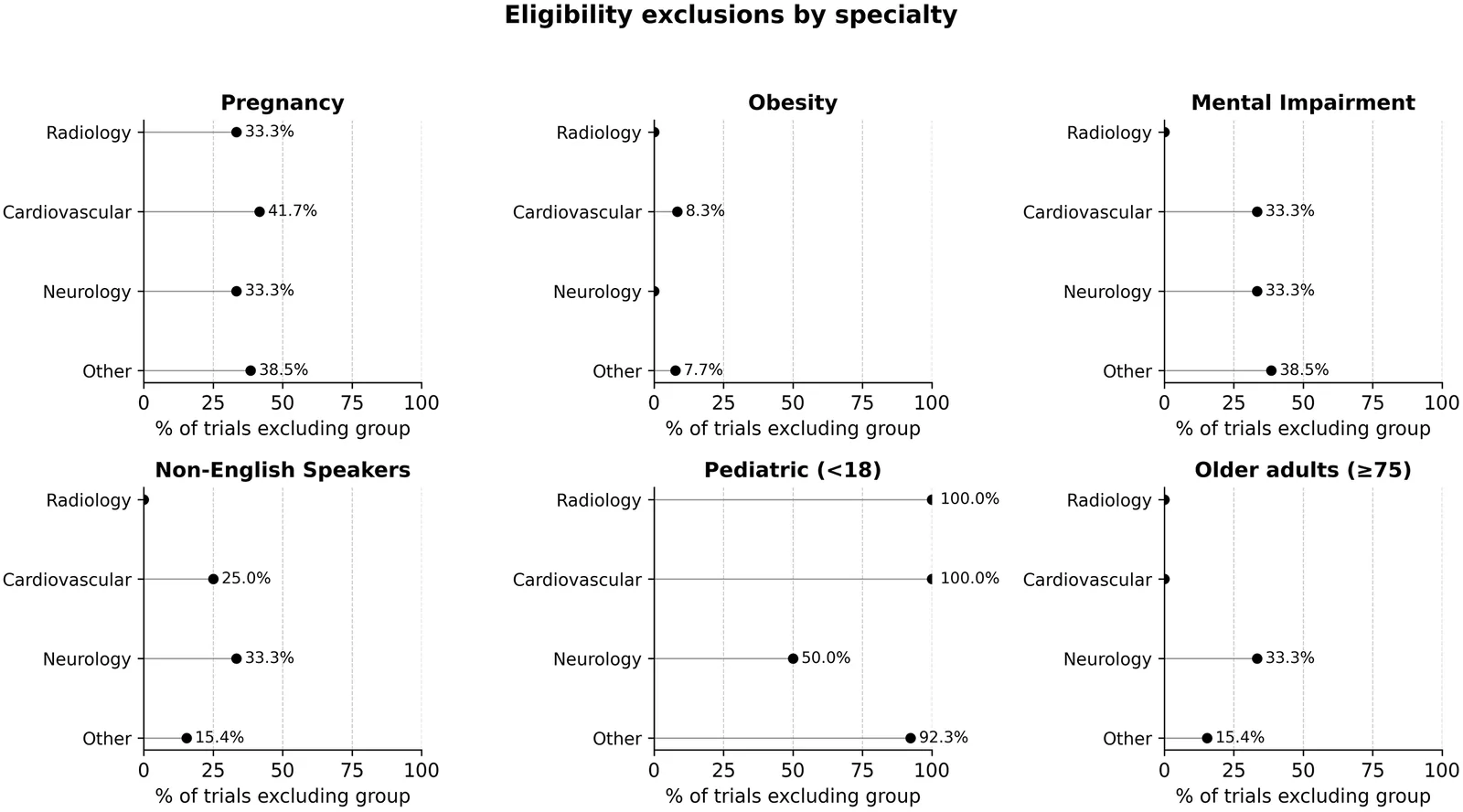
Eligibility exclusions by specialty. Points show the percentage of FDA-registered clinical trials for AI devices that excluded specific patient groups, stratified by specialty (Radiology *n* = 3, Cardiovascular *n* = 12, Neurology *n* = 6, Other *n* = 13). Exclusions based on age thresholds (pediatric (<18) and older adults (≥75)) are shown alongside other common criteria such as pregnancy, obesity, mental impairment, and non-English speakers, highlighting systematic exclusion of vulnerable populations.

Even where trials exist, their design raises concerns for clinical translation (Table 2). Nearly three-quarters enrolled fewer than 500 participants, and one-quarter included fewer than 100. Most were conducted exclusively in the United States (68%), limiting generalizability to international health systems. Of the 34 registered trials, 9 (27%) reported any subgroup analysis. These most commonly included sex (*n* = 5) and age (*n* = 4), while race or ethnicity was reported in only 3 trials and language in none. Reporting across demographic domains was incomplete and inconsistent, leaving major gaps in understanding differential performance across demographic groups. Systematic exclusions were also common (Fig 2): pregnancy exclusions were frequent in cardiovascular (42%) and radiology (33%) trials, and non-English speakers and patients with mental impairment were also frequently omitted, particularly in neurology. Pediatric populations were almost universally excluded, while exclusion of older adults was less common but notable in some specialties such as neurology. These patterns create a fundamental mismatch between trial populations and real-world patients, narrowing the evidence base and raising equity concerns [18].

**Fig 3:**
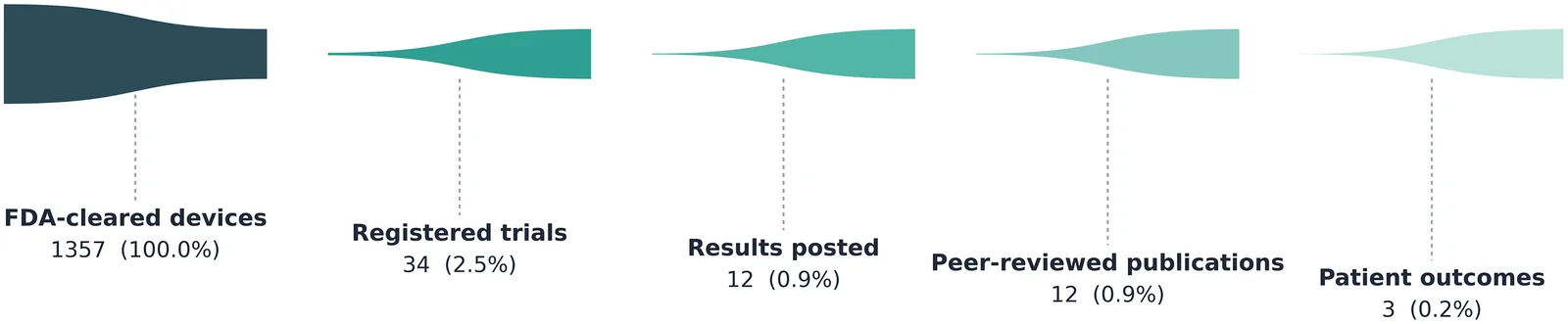
Funnel diagram showing attrition of clinical evidence for FDA-cleared AI devices. Each stage displays the count and percentage relative to all cleared devices (1,357). Tapered widths reflect the shrinking number of devices with registered trials, results posted, peer-reviewed publications, and patient outcomes.

Selective reporting further skews the literature. Of 1,357 FDA-cleared AI devices, only 34 (2.5%) had registered clinical trials. Among these devices, just 12 (0.9%) had results posted to ClinicalTrials.gov and 12 (0.9%) progressed to peer-reviewed publication, with only 3 (0.2%) reporting patient outcomes (Fig 3). Negative or inconclusive findings are seldom disseminated, reinforcing a literature dominated by algorithmic success [19].

Sponsorship patterns reinforce this bias: of the 34 trials, 32 (94%) were industry-led, with designs heavily focused on accuracy metrics rather than patient outcomes. For clinicians and health systems, this creates an evidence base that is incomplete, systematically biased, and tilted toward optimism.

## The risk of deploying untested devices

The limited clinical evidence for AI medical devices creates risks when these tools are deployed across diverse patient populations. While FDA clearance through the 510(k) pathway requires some testing, its “substantial equivalence” standard falls short of predicting real-world performance. Most devices are tested on the bench, but never in the field. The consequences of these gaps are not abstract. Devices excluded from validation in pregnant women, non-English speakers, or patients with mental health conditions may still be deployed in emergency or primary care settings where accurate diagnosis is critical. For instance, imaging and cardiac monitoring devices often excluded pregnant women from validation due to safety concerns or physiologic variability, yet these same tools may be used in obstetric emergencies where accurate diagnosis is vital. Geographic bias compounds these risks: most trials were confined to single academic centers or highly resourced health systems, leaving performance in community hospitals, rural clinics, and resource-constrained settings largely unknown. Without standardized demographic reporting, transparent methods, and open datasets, inequitable performance is both predictable and difficult to detect or correct [18,19]. Crucially, the 34 devices with registered trials represent only those companies that opted for transparency. Even within this more transparent subset, the patterns described above reveal an evidence base that is not merely limited but systematically shaped: dominated by industry sponsors, focused on surrogate endpoints, and largely silent on how devices perform across diverse populations. These patterns suggest that evidence for AI medical devices is not only scarce but selectively generated, raising concern about blind spots among the 1,323 devices with no registered trials at all. To be clear, the absence of published clinical evidence does not necessarily indicate deficient regulatory oversight; the FDA maintains non-public monitoring mechanisms, including manufacturer reporting requirements and the MAUDE adverse event database, that provide some degree of post-market surveillance. However, the lack of publicly accessible, peer-reviewed outcome data means that clinicians, health systems, and patients cannot independently assess whether a device improves care—a transparency gap that is distinct from, but compounded by, regulatory design choices. Without prospective, transparent, and inclusive evaluation, claims of transformative benefit will remain aspirational rather than evidence-based.

## Structural barriers to validation

The scarcity of patient-centered evidence for AI devices is not accidental, but the predictable outcome of structural barriers that consistently tilt innovation toward speed rather than rigor. While empirical ranking of these barriers (e.g., through Delphi panels or cost modeling) represents a valuable direction for future research, we present them here as interconnected and mutually reinforcing rather than hierarchically ordered, as their relative weight likely varies by device type, clinical specialty, and regulatory jurisdiction. These structural dynamics are reflected in the empirical patterns observed in our dataset, including the predominance of the 510(k) pathway among AI clearances and the extremely limited proportion of devices supported by prospective patient-centered trials. Economic pressures shape the incentives for developers, who gain reputational and financial advantages by reaching the market quickly, while the costs and uncertainties of prospective trials offer little immediate return [20,21]. AI healthcare startups, in particular, typically operate on compressed funding cycles in which investor valuations hinge far more on regulatory milestones such as FDA clearance than on evidence of clinical benefit, leaving patient outcomes peripheral to early-stage success [22–24]. This imbalance is further reinforced by reimbursement policies that provide coverage for FDA-cleared devices regardless of outcomes evidence, creating revenue streams that reward approval status over patient impact [25–27]. In such an environment, commercial success is tied to speed, not safety. Regulation amplifies this misalignment. Unlike pharmaceuticals, which must progress through phased trials to establish safety and efficacy, AI tools can gain clearance through the 510(k) pathway by demonstrating “substantial equivalence” to earlier devices, many of which themselves lack rigorous validation [8]. This predicate-based approach allows evidence gaps to cascade across generations of products. Current FDA guidance recommends, but does not require, prospective trials even for high-risk technologies, producing a dangerous asymmetry between regulatory approval and proof of clinical value [28,29]. The disparity in evidentiary standards across product types is stark. New pharmaceuticals approved through the FDA’s New Drug Application (NDA) pathway require pre-market clinical trial evidence demonstrating safety and effectiveness under the statutory “substantial evidence” standard [30], typically supported by large Phase III randomized controlled trials with pre-specified primary efficacy endpoints [31]. Similarly, high-risk Class III devices submitted via the Premarket Approval (PMA) pathway must provide clinical evidence of safety and effectiveness prior to approval [32]. By contrast, the majority of AI/ML-enabled devices reach the market through the 510(k) pathway, which does not require independent prospective clinical trial data if substantial equivalence to a predicate device can be demonstrated [8]. In our analysis, only 2.5% of cleared AI devices had any registered prospective clinical trial, highlighting the contrast in evidentiary expectations. While evidence gaps are not unique to AI (limitations in clinical evidence generation for traditional medical devices have also been recognized [33–35]), the scale and speed of AI device clearances (1,357 in around a decade), combined with their expanding clinical reach, make the absence of patient-centered outcome evidence for AI devices a uniquely pressing concern. Even when developers seek to pursue rigorous validation, they face formidable logistical obstacles. Prospective, multi-center studies for AI devices are more complex than traditional trials because they must be embedded within real clinical workflows, linked to heterogeneous electronic health record systems, and accompanied by provider training [20,36]. Coordinating such studies across diverse sites requires harmonized data standards and outcome definitions that remain rare, while the rapid pace of software iteration means that by the time a trial concludes, the version under evaluation may already be obsolete. These realities further discourage companies from investing in the type of evidence that clinicians and patients most need. The risks are magnified in resource-limited settings, where imported AI tools are often deployed without local validation. Many low- and middle-income countries lack the regulatory infrastructure to independently assess AI technologies, and thus rely on approval decisions made in high-income contexts [37]. Yet, algorithms trained predominantly on data from wealthier populations may underperform when applied to patients with different demographics, disease patterns, or health system constraints. Without mechanisms for local validation, such deployments risk reinforcing inequities and exposing vulnerable populations to tools whose reliability remains untested. Overcoming these barriers will require a fundamental shift in policy design. Just as drug development follows a structured progression from early safety studies to large-scale efficacy trials, AI devices should be subject to staged requirements that move from preliminary observational studies to prospective outcome validation and subgroup analyses before widespread deployment. Without such guardrails, marketing narratives will continue to outpace clinical evidence, and the cycle of innovation without accountability will persist.

## A roadmap for responsible AI evidence

Closing the gap between AI hype and patient benefit requires more than technical innovation; it demands a fundamental reorientation of evidence standards, incentives, and governance. Progress in this field should not be measured by the speed with which new tools reach the market, but by the clarity, durability, and inclusivity of the evidence that underpins their use. A credible roadmap must therefore involve not only developers but also clinicians, patients, policymakers, and regulators who share responsibility for ensuring that AI innovations serve health rather than headlines. With that shared premise, we can build on what already exists. Current regulatory scaffolding covers four areas: (1) safe iteration of learning systems [38,39]; (2) development and transparency practices for design, labeling, and user information [40,41]; (3) common baselines for clinical evaluation and software characterization [42,43]; and (4) lifecycle oversight that links submissions to post-market monitoring and aligns device law with AI obligations as they phase in [44,45]. What none of this yet requires is what our analysis shows is missing: prospective, pre-registered trials demonstrating patient outcomes across diverse sites and subgroups. The first step is transparency. Every patient-facing AI evaluation should be prospectively registered with a publicly accessible protocol and analysis plan, much like clinical trials listed on ClinicalTrials.gov. Journals and funders should enforce adherence to reporting standards such as SPIRIT-AI and CONSORT-AI, which require disclosure of outcomes, data sources, and limitations. Without such requirements, negative or inconclusive findings will remain hidden, reinforcing the literature’s positivity bias and giving clinicians an artificially optimistic view of AI performance [18]. Evidence generation should also be staged, reflecting the graduated approach used in drug development. We propose a three-phase framework: Phase 0 (pre-clearance) should include retrospective validation on diverse, representative datasets with mandatory demographic reporting. It addresses the first failure point: the absence of any pre-clearance evidence standard. Currently, 97.5% of cleared devices enter clinical use without a single registered prospective trial. Phase 0 would require retrospective validation on diverse, representative datasets with mandatory demographic reporting before clearance is granted, establishing a minimum evidential floor analogous to pre-investigational requirements in existing FDA device guidance [28,29]. Phase 1 (peri-clearance) should require singleor multi-site prospective studies (*n* ≥ 500) embedded in real clinical workflows, with pre-specified safety and usability endpoints. This phase addresses the second failure point: the inadequacy of existing trials for detecting clinically meaningful effects. Among the 34 registered trials identified, 73.5% enrolled fewer than 500 participants, sample sizes too small to power subgroup analyses or detect differential performance across demographic groups. The *n* ≥ 500 threshold for Phase 1 is therefore not an arbitrary standard but the level at which the majority of current trials already fall short. These would be single- or multi-site prospective studies embedded in real clinical workflows, with pre-specified safety and usability endpoints. Funding mechanisms such as the NIH AI-TRAC program and FDA regulatory science grants can support this phase. Finally, Phase 2 (post-clearance) should mandate multi-center outcome trials (*n* ≥ 2,000) with patient-centered endpoints and pre-specified subgroup analyses across equity-relevant strata. This addresses the terminal failure point: the near-complete absence of patient-centered outcome data. Only 3 of 1,357 cleared devices (0.2%) have been evaluated for outcomes that matter to patients (mortality, morbidity, or readmissions). Closing this gap requires multi-center trials of sufficient scale (*n* ≥ 2,000) to detect differences in patient-centered endpoints across equity-relevant subgroups, reflecting the evidential standard established for pharmaceuticals under the FDA’s New Drug Application pathway [30,31]. Before large-scale outcome trials, developers should conduct early, workflow-embedded evaluations that test usability, capture safety signals, and identify integration challenges. These studies should explicitly involve patients and clinicians in design and evaluation, ensuring that tools address genuine needs rather than reinforcing inefficiencies or disparities [46]. Only after such phased evaluations should broader trials proceed, measuring patient-centered outcomes like mortality, morbidity, length of stay, and quality of life. These studies must also assess subgroup performance across sex, age, race, language, comorbidities, and geography, ensuring equitable benefit across diverse populations [18,19]. Because AI is not static, governance must extend beyond approval. Regulators and health systems should require pre-specified change-control plans, strict versioning, and post-market surveillance that includes drift detection, bias monitoring, and re-validation triggers. The FDA’s Predetermined Change Control Plan [47] offers a starting point, but health systems can go further by creating institutional AI formularies that vet tools against local standards and track performance over time. Shared governance mechanisms, where regulators, health systems, academia, industry, and civil society co-develop standards for transparency, accountability, and equity, are essential to align incentives and prevent the unchecked spread of under-validated tools [48]. Global deployment adds another layer of responsibility. AI devices validated in high-income countries cannot simply be exported to low- and middle-income settings without context-specific testing. Disease prevalence, clinical workflows, infrastructure, and population genetics differ significantly, and tools trained elsewhere may fail when deployed abroad. International standards, including those proposed by the WHO [49], should require local validation and equity safeguards before widespread adoption, ensuring that global health systems are not treated as testing grounds for under-validated technologies. Finally, incentives must be realigned to reward rigor. Funders and journals can prioritize validation studies, particularly those reporting null or negative findings. Insurers and health technology assessment bodies can tie reimbursement to demonstrated clinical benefit, rather than regulatory status alone. Professional liability frameworks should also evolve, making clear that deploying inadequately validated tools carries risks not only for patients but also for providers and institutions. By aligning financial, professional, and regulatory incentives with robust evidence, the system can encourage companies to invest in the trials that truly matter. Readiness should no longer mean regulatory clearance or technical novelty; it should mean demonstrated, durable, and equitable benefit for patients in the settings where they receive care. Only by shifting incentives from speed to accountability, from novelty to clinical durability, and from marketing claims to measurable patient outcomes can AI fulfill its promise as a transformative force in medicine.

## Global implications and ethical risks

FDA clearance often functions as a de facto passport for international commercialization of AI medical devices. Developers routinely leverage U.S. authorization to market tools abroad, including in low- and middle-income countries where regulatory infrastructures are less mature and contextual validation is sparse [8]. A systematic review of AI deployments in low- and middle-income country health systems reveals troubling patterns of unreliability, limited adaptability to local workflows, and opaque reporting of training datasets [50]. Concrete examples illustrate these risks: IBM Watson for Oncology, initially cleared in the U.S. and subsequently deployed in India, China, and Denmark, showed concordance with expert recommendations as low as 12% for gastric cancer in Chinese populations and 33% in Danish cohorts [13,14]—markedly below performance reported during initial validation. This pattern creates a dual hazard. On one hand, low- and middle-income countries risk becoming inadvertent testing grounds for under-validated technologies, amplifying disparities in safety, access, and outcomes. On the other hand, the absence of robust post-market surveillance means deficiencies in effectiveness may remain undetected until harm has already occurred. Scholars have described this dynamic as a form of “algorithmic colonialism,” where technologies developed in high-resource settings are exported without meaningful local engagement or adaptation [51,52]. Underlying these risks are persistent biases in training datasets, which remain heavily skewed toward high-income populations. AI systems trained in such contexts may underperform when applied to patients with different genetic backgrounds, disease prevalence, or health system constraints. Infrastructure gaps, from limited broadband connectivity to shortages of trained personnel, further hinder safe deployment and may render tools unreliable in precisely the settings where they are most needed [50]. Recent WHO regulatory guidance emphasizes the importance of clearly specified intended use, analytical and clinical validation on representative external datasets, and strengthened post-market monitoring [53]. These recommendations align with broader WHO calls for harmonized international standards that mandate local validation, transparent demographic reporting, and context-specific safety requirements [49]. Ethical AI, therefore, must be both globally informed and locally accountable: validated not only in well-resourced academic centers but also in the environments where patients will actually receive care. Without such safeguards, technologies intended to democratize healthcare risk entrenching inequities on a global scale.

## Limitations

Several limitations should be acknowledged. First, our analysis relies on publicly available registries (ClinicalTrials.gov, PubMed, FDA device database) and may undercount proprietary validation studies conducted internally by manufacturers but not publicly disclosed. Second, not all FDA 510(k) summary pages were accessible in full text, potentially leading to missed trial linkages. Third, our registry-based approach focused on prospectively registered trials and did not systematically search for unregistered validation studies that may exist in the published literature. This approach was deliberately chosen to assess transparency and prospective trial registration practices, but may not capture the full scope of AI device validation efforts. Fourth, the absence of a non-AI device comparator group limits our ability to determine whether the evidence gaps identified are unique to AI/ML-enabled devices or reflect broader patterns in medical device regulation. Fifth, device-level classification by FDA class (I, II, III) and regulatory pathway (510(k), De Novo, PMA), degree of AI autonomy (assistive vs autonomous), and level of physician oversight was not systematically extracted for all 1,357 devices, limiting risk-stratified analyses. Finally, rapid evolution of the FDA database means that new clearances and trial registrations occurring after our December 5, 2025 cutoff are not captured.

## Conclusion

AI tools must be life-tested before they can be called life-saving. Regulatory clearance has far outpaced clinical validation, creating an ecosystem where innovation advances without accountability and patients bear the risks of unproven technologies. Among 1,357 cleared devices, only three have been evaluated for patient-centered outcomes such as mortality, morbidity, or readmissions, and even these studies relied on modest sample sizes and limited geographic diversity. The path forward requires three commitments, each tied to responsible actors: evidence standards that match the stakes through prospective trials measuring patient outcomes rather than algorithmic accuracy, with the FDA mandating pre-registration of prospective trials for all Class II and III AI devices before clearance; validation in the populations and contexts where tools will actually be used, from academic centers to community clinics and resource-limited health systems, with the WHO requiring local validation and equity safeguards before international deployment; and transparency that enables clinicians, patients, and policymakers to make informed choices about both benefits and risks, with CMS and insurers tying reimbursement to demonstrated clinical benefit rather than regulatory status alone. This is not a call to slow innovation but to make it count, for the current trajectory risks turning medicine into a fail-fast marketplace where algorithms are introduced for novelty, rapidly adopted, and quietly discarded while patients absorb the costs of uncertainty. The infrastructure for reform, such as FDA frameworks, international WHO guidance, and digital health pathways, already exists and provides the scaffolding. However, the will to require proof that these tools truly help the patients who need the most is absent. Progress will not be measured by the number of FDA-cleared algorithms, but by whether people live healthier lives, and whether those benefits are shared equitably across all populations. The central challenge is not primarily technical, but institutional: regulatory acceleration without corresponding clinical accountability leaves patients to absorb the consequences of an evidence gap they did not create.

### Ethics statement

This study was based entirely on publicly available data and did not involve human subjects research. All data were obtained from publicly accessible databases including the U.S. Food and Drug Administration (FDA) medical device database, the American College of Radiology

Data Science Institute (ACR DSI) AI Central catalogue, ClinicalTrials.gov, and PubMed. No institutional review board approval or informed consent was required as no patient data, private information, or human subjects were involved in this analysis.

## Supporting information

S1 Data Trial-level extraction dataset. Standardized trial-level data extracted from ClinicalTrials.gov registry records for clinical trials linked to FDA-cleared AI/ML-enabled medical devices, including trial identifier (NCT ID), medical specialty, clinical task, workflow integration point, developer type, validation type, study design, sample size, geographic locations, patient age range, and eligibility exclusions, together with the reference sheet and category definitions used for extraction.
(XLSX)

S1 File Supplementary materials. Supplementary materials containing extended methods and results: study design and scope (S-01), device identification and deduplication (S-02), the three-phase evidence mapping approach (S-03), eligibility criteria and manual extraction (S-04), the outcome classification framework (S-05), regulatory pathway breakdown (S-06), supplementary results (S-07), trial-level characteristics (S-08), eligibility exclusions by specialty (S-09), bias and equity concerns by trial (S-10), subgroup analyses reporting (S-11), and search strategy and code availability (S-12).
(PDF)
