# Supplementary material for: 1,357 AI medical devices cleared, 3 actually tested on patient outcomes
Source: PLOS Digit Health. 2026 Aug 19;5(8):e0001597. doi: 10.1371/journal.pdig.0001597 (PMC13489462; doi:10.1371/journal.pdig.0001597)
Supplement: Supplementary file 1 [file pdig.0001597.s002.pdf]

Supplementary Materials

1,357 AI Medical Devices Cleared, 3 Actually Tested on Patient Outcomes

PLOS Digital Health

Contents

1 S-01: Study Design and Scope **3**

2 S-02: Device Identification and Deduplication **3**

2.1 Data Sources 3

2.2 Deduplication Procedure 4

3 S-03: Three-Phase Evidence Mapping Approach **4**

3.1 Phase 1: Automated PubMed Search 4

3.2 Phase 2: ClinicalTrials.gov Identifier Extraction 5

3.3 Phase 3: Publication Linkage 5

4 S-04: Eligibility Criteria and Manual Extraction **5**

4.1 Trial Inclusion Criteria 5

4.2 Data Extraction 6

5 S-05: Outcome Classification Framework **6**

5.1 Patient-Centered Outcomes 7

5.2 Process Endpoints 7

5.3 Surrogate/Diagnostic Accuracy Endpoints 7

5.4 Rationale 8

6 S-06: Regulatory Pathway Breakdown **8**

7 S-07: Supplementary Results **9**

7.1 Overview 9

7.2 Trials with Patient-Centered Outcomes 9

8 S-08: Trial-Level Characteristics **10**

9 S-09: Eligibility Exclusions by Specialty **13**

10 S-10: Bias and Equity Concerns by Trial **13**

11 S-11: Subgroup Analyses Reporting **19**

12 S-12: Search Strategy and Code Availability **20**

1 S-01: Study Design and Scope

This study was designed as a regulatory evidence census and structured evidence-mapping analysis of all publicly available validation signals for FDA-cleared or approved AI/ML-enabled medical devices, rather than as a PRISMA-style meta-analysis of treatment effects. The objective was to characterize the presence, design features, and transparency of prospective clinical validation linked to authorized devices. Trials were included regardless of publication or results posting status. This inclusive approach was chosen to assess the full landscape of prospective trial registration practices linked to FDA-authorized AI/ML devices and to avoid survivorship bias toward published-only studies.

Terminology. Throughout this supplement and the main manuscript, “FDA-cleared” refers to devices authorized via the 510(k) pathway, “FDA-approved” refers to those authorized via Premarket Approval (PMA), and “FDA-authorized” or “cleared or approved” is used as an umbrella term encompassing 510(k), De Novo, and PMA pathways. The vast majority of AI/ML-enabled devices have reached the market through the 510(k) pathway.

2 S-02: Device Identification and Deduplication

2.1 Data Sources

All AI/ML-enabled medical devices listed in the following two sources as of December 5, 2025 were included:

1. **FDA Public Device Database:** The U.S. Food and Drug Administration’s publicly available list of AI/ML-enabled medical devices, which catalogues devices that have received marketing authorization (510(k) clearance, De Novo classification, or PMA approval).

2. **ACR Data Science Institute (DSI) AI Central Catalogue:** A curated registry of AI-based tools in medical imaging maintained by the American College of Radiology.

2.2 Deduplication Procedure

Devices appearing in both databases were deduplicated using the following identifiers:

- Manufacturer name (standardized for variations in corporate naming, e.g., abbreviations, parent company names)
- Device trade name
- FDA submission identifiers: 510(k) number, De Novo number, or PMA number

Duplicate entries were defined as records sharing the same manufacturer and device name with matching FDA submission identifiers. When a single device held multiple FDA submissions (e.g., an initial 510(k) clearance followed by a supplemental submission), each unique submission was counted once. Devices with distinct trade names but shared underlying technology from the same manufacturer were retained as separate entries if they held separate FDA submission numbers.

After deduplication, 1,357 unique AI/ML-enabled medical devices were identified.

3 S-03: Three-Phase Evidence Mapping Approach

Evidence mapping proceeded in three sequential phases:

3.1 Phase 1: Automated PubMed Search

An automated search of PubMed was conducted using device name variants and manufacturer identifiers combined with predefined patient-outcome terms. Search terms included:

- Device-specific terms: device trade name, manufacturer name, and known name variants

- Patient-outcome terms: mortality, morbidity, length of stay, readmission, complications, survival, adverse events, quality of life, functional status

Results were screened for relevance to clinical outcome evaluation of FDA-authorized AI/ML devices.

3.2 Phase 2: ClinicalTrials.gov Identifier Extraction

ClinicalTrials.gov identifiers (NCT numbers) were systematically extracted from FDA 510(k) summary pages using automated data retrieval with scraping. Each 510(k) summary page was parsed for embedded links or text references to ClinicalTrials.gov registrations. Extracted NCT identifiers were cross-referenced against the ClinicalTrials.gov registry to confirm validity and retrieve trial metadata.

3.3 Phase 3: Publication Linkage

Identified NCT registrations were linked to peer-reviewed publications using:

- PubMed secondary identifier searches (NCT[si]) to identify publications citing specific trial registration numbers
- Abstract retrieval and manual review to confirm linkage between trial registrations and published studies

4 S-04: Eligibility Criteria and Manual Extraction

4.1 Trial Inclusion Criteria

Trials were included if they met the following criteria:

- Prospectively registered on ClinicalTrials.gov
- Linked to an FDA-cleared or approved AI/ML-enabled medical device

- Regardless of publication status, results posting status, or completion status

4.2 Data Extraction

For all eligible trials (n=34), two independent reviewers manually extracted the following variables from ClinicalTrials.gov registry records:

- **Study characteristics:** Study title, NCT identifier, study design (observational, interventional, other), validation type (prospective, retrospective, mixed)
- **Clinical context:** Medical field/specialty, clinical task (diagnostic, screening, therapeutic/treatment guidance, predictive, other), workflow integration point (pre-visit, imaging-time, intra-procedural, post-scan, not specified)
- **Population:** Sample size, geographic location(s), patient age range
- **Exclusion criteria:** Pregnancy, obesity, mental impairment, non-English speakers, other specified exclusions
- **Reporting:** Subgroup analysis reported (yes/no), bias/equity concerns (free text), results submitted to ClinicalTrials.gov, peer-reviewed publication identified, patient-centered outcome assessed
- **Sponsorship:** Developer type (industry, academic, partnership, government/NIH, unknown)

When available, data were supplemented with information from peer-reviewed publications and posted trial results. Discrepancies between reviewers were resolved by consensus.

5 S-05: Outcome Classification Framework

Primary endpoints were classified a priori into three categories based on the trial's stated primary endpoint:

5.1 Patient-Centered Outcomes

Outcomes were classified as patient-centered when the primary endpoint directly reflected tangible benefit to patients' survival, safety, well-being, or functional capacity. These included:

- **Primary (hard clinical) outcomes:** Mortality (e.g., survival, death rates) and major morbidity (e.g., stroke, myocardial infarction, serious adverse events)
- **Hospitalizations and readmissions:** When designated as primary endpoints
- **Quality-of-life measures:** Validated instruments such as SF-36, EQ-5D
- **Functional status:** Activities of daily living, disability scores, return to work
- **Symptom burden:** Measures affecting daily life

5.2 Process Endpoints

Process measures such as workflow efficiency, time savings, or length of stay without direct linkage to clinical events were classified as process endpoints and were not considered patient-centered.

5.3 Surrogate/Diagnostic Accuracy Endpoints

The following were classified as surrogate endpoints and not considered patient-centered:

- Diagnostic accuracy metrics (e.g., sensitivity, specificity, AUC, positive/negative predictive value)
- Technical performance metrics (e.g., image quality, signal detection)
- Physiological measurements without linkage to clinical outcomes
- Specimen collection or analytical validation

5.4 Rationale

Because the aim of this study was to characterize validation presence and design features rather than to estimate pooled clinical effects, formal risk-of-bias scoring tools (e.g., ROBINS-I, QUADAS-AI) were not applied.

6 S-06: Regulatory Pathway Breakdown

Table 1 provides the distribution of 1,357 FDA-authorized AI/ML-enabled medical devices by regulatory pathway as of December 5, 2025.

Table 1: Distribution of FDA-authorized AI/ML-enabled medical devices by regulatory pathway (as of December 5, 2025).

Regulatory Pathway	n	%
510(k) Clearance	1,317	97.1
De Novo Classification	34	2.5
Premarket Approval (PMA)	6	0.4
**Total**	**1,357**	**100.0**

The 510(k) pathway accounted for the vast majority of AI/ML device authorizations. Unlike the De Novo pathway, which requires independent demonstration of safety and effectiveness for novel devices without a suitable predicate, the 510(k) route permits marketing authorization based on "substantial equivalence" to a predicate device, allowing evidence gaps to propagate through chains of predicate devices. Premarket Approval (PMA) applies to the highest-risk (Class III) devices and requires the most rigorous clinical evidence prior to authorization.

7 S-07: Supplementary Results

7.1 Overview

Of 1,357 FDA-authorized AI/ML-enabled medical devices as of December 5, 2025, 34 (2.5%) were linked to registered prospective clinical trials on ClinicalTrials.gov. Among these, 12 (0.9%) had results posted to ClinicalTrials.gov, 12 (0.9%) had peer-reviewed publications identified, and only 3 (0.2%) evaluated patient-centered outcomes such as mortality, morbidity, or readmissions.

7.2 Trials with Patient-Centered Outcomes

Three trials were identified as evaluating patient-centered outcomes:

1. **NCT02391987** — Integrated Tele-monitoring and Patient-centric Health Coaching Strategy in Patients Hospitalized With Heart Failure. Cardiovascular; therapeutic application. Randomized controlled trial (n=112) across 5 U.S. locations evaluating tele-monitoring and health coaching to reduce readmissions post-discharge for acute decompensated heart failure.

2. **NCT01189331** — CT Coronary Angiography and Computational Fluid Dynamics (DISCOVER-FLOW Study). Cardiovascular; diagnostic application. Prospective multicenter observational study (n=103) across South Korea, Latvia, and the United States comparing CT-derived fractional flow reserve (FFR_{CT}) with invasive FFR for diagnosis of lesion-specific ischemia.

3. **NCT06235190** — Assessment of the Safety and Efficacy of the Felix NeuroAI Wristband in Essential Tremor (TRANQUIL). Neurology; therapeutic/treatment guidance. Randomized controlled trial (n=126) across 8 U.S. and 4 China locations evaluating a wearable AI-based therapeutic device for essential tremor.

8 S-08: Trial-Level Characteristics

Table 2 presents detailed characteristics of all 34 registered clinical trials linked to FDA-authorized AI/ML-enabled devices.

Table 2: Characteristics of registered clinical trials linked to FDA-authorized AI/ML-enabled devices.

NCT ID	Specialty	Clinical Task	Design	Valid. Type	Dev. Type	Sample Size	Location(s)	Results	Pub.	Pt. Out.
NCT04702451	Cardiovascular	Therapeutic	Interventional	Prospective	Industry	85	US, Belgium, France, Germany, Netherlands	Yes	Yes	No
NCT06460246	Other	Diagnostic	Interventional	Prospective	Industry	12	US	No	No	No
NCT06351878	Other	Screening	Observational	Prospective	Industry	200	US	No	No	No
NCT03606382	Other	Diagnostic	Observational	Prospective	Industry	6	US	No	Yes	No
NCT05853380	Other	Diagnostic	Observational	Prospective	Industry	85	US	Yes	No	No
NCT06027164	Cardiovascular	Diagnostic	Observational	Prospective	Industry	153	US	No	No	No
NCT06070389	Other	Diagnostic	Interventional	Prospective	Industry	533	US	No	Yes	No
NCT05857943	Other	Screening	Interventional	Prospective	Industry	363	US	No	No	No
NCT03207685	Neurology	Screening	Observational	Prospective	Industry	100	US	No	No	No
NCT03583957	Neurology	Screening	Observational	Prospective	Industry	750	US	No	No	No
NCT05266235	Cardiovascular	Screening	Observational	Prospective	Industry	274	Belgium, France	No	No	No
NCT03112005	Other	Screening	Observational	Prospective	Industry	940	US	No	No	No
NCT04885062	Other	Diagnostic	Interventional	Prospective	Partnership	100	US	Yes	No	No
NCT04612868	Other	Diagnostic	Observational	Prospective	Industry	462	US, Israel	Yes	No	No
NCT04546763	Cardiovascular	Diagnostic	Observational	Retrospective	Industry	117	US	No	Yes	No
NCT04626271	Other	Diagnostic	Other	Prospective	Industry	250	US	Yes	No	No
NCT03469986	Neurology	Diagnostic	Interventional	Prospective	Industry	505	US	No	Yes	No
NCT04828590	Cardiovascular	Diagnostic	Observational	Retrospective	Industry	302	US, Austria, France, Italy, Poland	Yes	No	No
NCT04699812	Cardiovascular	Screening	Other	Prospective	Industry	573	US	Yes	No	No
NCT04074434	Cardiovascular	Diagnostic	Observational	Prospective	Industry	160	US	No	No	No
NCT03595501	Other	Diagnostic	Observational	Prospective	Industry	679	US	No	No	No
NCT02477943	Neurology	Other	Observational	Prospective	Industry	520	US	No	Yes	No

Continued on next page

Table 2: (continued)

NCT ID	Specialty	Clinical Task	Design	Valid. Type	Dev. Type	Sample Size	Location(s)	Results	Pub.	Pt. Out.
NCT02391987	Cardiovascular	Other	Interventional	Prospective	Academic	112	US	Yes	No	Yes
NCT01308164	Other	Therapeutic	Interventional	Prospective	Industry	25	Israel, Slovenia	No	No	No
NCT02902328	Radiology	Other	Observational	Prospective	Industry	30	Not stated	No	No	No
NCT02143102	Cardiovascular	Diagnostic	Observational	Prospective	Industry	125	US	Yes	Yes	No
NCT03226262	Cardiovascular	Diagnostic	Observational	Prospective	Industry	301	US, Belgium, Denmark, Israel	Yes	Yes	No
NCT01189331	Cardiovascular	Diagnostic	Observational	Prospective	Industry	103	South Korea, Latvia, US	No	Yes	Yes
NCT02367300	Neurology	Diagnostic	Observational	Prospective	Industry	981	US	No	Yes	No
NCT02440139	Radiology	Diagnostic	Other	Retrospective	Industry	12	US	Yes	Yes	No
NCT01261507	Radiology	Diagnostic	Observational	Retrospective	Industry	15	US	Yes	No	No
NCT05316350	Cardiovascular	Diagnostic	Interventional	Prospective	Industry	685	US, France	No	No	No
NCT05455281	Other	Predictive	Observational	Prospective	Industry	55	US, Israel	No	No	No
NCT06235190	Neurology	Therapeutic	Interventional	Prospective	Industry	126	US, China	No	Yes	Yes

Legend: Results = results posted to ClinicalTrials.gov; Pub. = peer-reviewed publication identified; Pt. Out. = patient-centered outcome assessed; Valid. Type = validation type; Dev. Type = developer type.

9 S-09: Eligibility Exclusions by Specialty

Table 3 summarizes the frequency of common eligibility exclusion criteria across registered clinical trials, stratified by medical specialty.

Table 3: Eligibility exclusion criteria by medical specialty among FDA-authorized AI/ML device trials (n=34).

Exclusion Criterion	Radiology (n=3)	Cardio. (n=12)	Neurology (n=6)	Other (n=13)	Overall (n=34)
Pregnancy	1 (33%)	5 (42%)	2 (33%)	5 (38%)	13 (38%)
Obesity	0 (0%)	1 (8%)	0 (0%)	1 (8%)	2 (6%)
Mental impairment	0 (0%)	4 (33%)	2 (33%)	5 (38%)	11 (32%)
Non-English speakers	0 (0%)	3 (25%)	2 (33%)	2 (15%)	7 (21%)
Pediatric (<18 years)	1 (33%)	12 (100%)	3 (50%)	12 (92%)	28 (82%)
Older adults (≥75 years)	1 (33%)	1 (8%)	1 (17%)	4 (31%)	7 (21%)

Values represent the number (and percentage) of trials within each specialty that explicitly excluded each population group. "Not stated" responses were not counted as exclusions.

10 S-10: Bias and Equity Concerns by Trial

For each registered trial, two independent reviewers assessed potential bias and equity concerns based on eligibility criteria, population representativeness, sponsorship, and study design limitations. A summary of key concerns is presented below.

Table 4: Reviewer-assessed bias and equity concerns for registered AI/ML device trials.

NCT ID	Bias/Equity Concerns
NCT04702451	Significant male overrepresentation (67% male vs 33% female) limits generalizability to women with persistent AF. Industry-sponsored study using proprietary AI technology may not generalize to centers without access to this specific software system.
NCT06460246	Very small sample size (n=12) limits generalizability. Industry-sponsored device validation study with short duration (3 days) may not reflect real-world performance across diverse populations.
NCT06351878	Single site study limits geographic and demographic diversity. Participants required to use a mobile app connected to a cloud-based AI system, raising potential bias related to digital literacy, smartphone ownership, and internet access.
NCT03606382	Extremely small sample size (n=6) severely limits generalizability. Single-site study with exclusion of those unable to understand instructions may bias against non-English speakers and populations with lower health literacy.
NCT05853380	Study intentionally included diverse skin tones (Fitzpatrick Scale 1–6) which supports equity. Industry sponsorship raises potential bias concerns.
NCT06027164	Single-site study limits geographic diversity. Language restrictions (English/Spanish only) exclude significant populations. Exclusion of patients with chest surgery/deformities removes populations who might particularly benefit from portable ultrasound access.

Continued on next page

Table 4: (continued)

NCT ID	Bias/Equity Concerns
NCT06070389	Two-site study limits geographic diversity. Extensive medical exclusions (COPD, CHF, neuromuscular disorders) exclude populations who would primarily benefit from home sleep testing devices. Requirement to read/understand consent may exclude non-English speakers.
NCT05857943	Exclusion criteria details not fully specified. Industry sponsorship noted.
NCT03207685	Pregnancy exclusion noted. Industry sponsorship.
NCT03583957	Broad age range (5 years and older) enhances generalizability, but focus on pediatric patients may limit generalizability to adults, particularly older adults with different seizure profiles or comorbidities.
NCT05266235	Does not specify exclusion criteria beyond age groups, potentially limiting generalizability. Lack of mention of common exclusions may imply broad inclusion but could overlook biases in recruitment from specific European hospitals.
NCT03112005	Subgroup analysis reported. Limited additional exclusion details available.
NCT04885062	Potential conflicts of interest: several authors are company employees. Research grants provided by the device manufacturer.
NCT04612868	Extensive exclusions eliminate many patients who would typically need diabetic retinopathy screening in clinical practice. Study population appears healthier than typical diabetic patients, potentially overestimating real-world diagnostic performance.

Continued on next page

Table 4: (continued)

NCT ID	Bias/Equity Concerns
NCT04546763	Language requirement excludes non-English speakers. Highly selected population (paroxysmal AF patients with specific documented AF burden criteria) may not represent typical patients.
NCT04626271	Excludes non-English speakers and those unfamiliar with smartphones, disproportionately affecting elderly patients, immigrants, and lower socioeconomic populations. Exclusion of visually impaired individuals limits accessibility.
NCT03469986	Excluding non-English speakers limits applicability in multilingual communities. Requiring normal hearing/vision and cooperation with seated eye-tracking excludes children with sensory impairments, motor/behavioral regulation challenges, or genetic conditions.
NCT04828590	Excludes patients with obesity (BMI >35), acute coronary syndromes, and prior cardiac interventions. International multi-center design helps broaden applicability.
NCT04699812	Exclusion of individuals with mental impairment, physical disability, and implantable devices restricts real-world generalizability. Older, multimorbid, device-dependent patients at highest AF risk may be underrepresented.
NCT04074434	Requires participants to read and speak English. Exclusion of patients with paced rhythms is clinically appropriate but may limit applicability to older populations with cardiac devices at higher risk for AF.

Continued on next page

Table 4: (continued)

NCT ID	Bias/Equity Concerns
NCT03595501	Low bias/equity concerns. Device validation study with primarily technical/procedural exclusions rather than patient-based characteristics. Accepts healthy volunteers and includes pediatric patients (≥3 months).
NCT02477943	Exclusion of non-English speakers creates a major barrier. Narrow age range (18–25 years, college athletes only) severely limits generalizability. Exclusion of individuals with mental health conditions or neurological history may not reflect real-world population.
NCT02391987	Exclusion of patients "not visually and tactile capable of smartphone and home device usage" creates significant barriers for elderly patients and those with disabilities. Requirement for "adequate cell phone coverage" may exclude rural and underserved communities.
NCT01308164	Exclusion of patients with "any significant diseases or conditions including psychiatric disorders and substance abuse" creates major barriers. Limitation to pediatric patients (10–18 years) in only two countries restricts generalizability.
NCT02902328	Minimal bias and equity concerns as primarily a technical validation study. Acceptance of healthy volunteers broadens participation. Lack of specified geographic locations makes it difficult to assess population diversity.

Continued on next page

Table 4: (continued)

NCT ID	Bias/Equity Concerns
NCT02143102	95% Hispanic/Latino population limits generalizability across racial/ethnic groups with different atherosclerotic disease patterns. Carotid endarterectomy population represents severe disease only; performance uncertain for earlier-stage plaques.
NCT03226262	Excluded patients with reduced ejection fraction (≤45%), recent acute coronary syndromes, and complex coronary anatomy, limiting generalizability. Geographic concentration in developed countries may not reflect global applicability.
NCT01189331	Excluded patients with significant arrhythmias, high heart rates, low blood pressure, and contraindications to commonly used cardiac medications. Exclusion of prior CABG patients represents a significant limitation.
NCT02367300	Excluded patients with multiple neurological and psychiatric conditions common in real-world emergency department populations, including dementia, Parkinson's disease, multiple sclerosis, seizure disorders, and substance dependence.
NCT02440139	Narrow age restriction (55–77 years). Exclusion of symptomatic patients limits applicability. Small sample of only 12 radiologists. Cases derived from National Lung Screen Trial may not reflect broader populations.
NCT01261507	Geographic restriction to Baltimore/DC metropolitan area. Exclusion of poor image quality cases may not reflect real clinical conditions. Small sample of 15 radiologists.

Continued on next page

Table 4: (continued)

NCT ID	Bias/Equity Concerns
NCT05316350	Industry-sponsored study (Withings). Wrist circumference requirement (140–210mm) may exclude patients at extremes of body size. Primarily US-based enrollment. Requires smartphone and digital literacy.
NCT05455281	Small sample size (n=55). Strict fertility criteria exclude older women and those with diminished ovarian reserve who may most benefit from AI-assisted embryo selection. Limited to 3 sites.
NCT06235190	Industry-sponsored with multi-region enrollment (US and China) providing geographic diversity. Double-blind, sham-controlled RCT design is rigorous. Excludes patients with prior treatments like deep brain stimulation.

11 S-11: Subgroup Analyses Reporting

Of 34 registered trials, 9 (26.5%) reported any subgroup analysis. These most commonly included:

- Sex: 5 trials
- Age: 4 trials
- Race/ethnicity: 3 trials
- Language: 0 trials

Reporting across demographic domains was incomplete and inconsistent, leaving major gaps in understanding differential device performance across demographic groups.

12 S-12: Search Strategy and Code Availability

The automated PubMed and ClinicalTrials.gov search strategies, data retrieval, and data extraction pipelines used in this study were implemented in Python. The full device list, trial identifiers, extraction outputs, and analysis code are available at: https://github.com/24rahul/FDA-devices

The FDA list of Artificial Intelligence-Enabled Medical Devices was queried on December 5, 2025, from: `https://www.fda.gov/medical-devices/software-medical-device-samd/artificial-intelligence-enabled-medical-devices`.

The complete dataset of FDA-authorized AI/ML-enabled devices with linked trial information is provided as a supplementary Excel file (FDA_AI_Trials_Eligibility_Analysis_Dec2025.xlsx). This file contains three worksheets: (1) **Main**, with trial-level extracted data for all 34 registered trials including NCT identifier, medical specialty, clinical task, workflow integration point, developer type, validation type, study design, sample size, geographic locations, patient age range, exclusion criteria (pregnancy, obesity, mental impairment, non-English speakers, and other), subgroup analysis reporting, bias and equity concerns, results submission status, peer-reviewed publication status, and patient-centered outcome assessment; (2) **Reference Sheet**, with field definitions and coding instructions for each extracted variable; and (3) **Categories**, with the standardized coding taxonomy used for medical specialty, clinical task, workflow integration, developer type, validation type, study design, and exclusion criteria.
